# A randomized controlled pilot trial comparing peri-implant tissue health beneath CAD/CAM-milled PEEK versus cobalt-chromium telescopic crowns for mandibular overdentures

**DOI:** 10.1186/s40729-026-00690-6

**Published:** 2026-05-13

**Authors:** Iman A. El Asfahani, Amr Khalifa, Doaa Tawfik, Emad Agamy, Mahmoud G. Salloum

**Affiliations:** 1 Department of Prosthodontics, Faculty of Dentistry, Minia University, Minia, Egypt; 2 Department of Prosthodontics Faculty of Dentistry, Alamein International University, Matrouh, Egypt; 3 Faculty of Dentistry, Sinai University - Arish Branch, Arish, Egypt; 4 Department of Prosthodontics, Faculty of Dentistry, Sinai University - Kantara Branch, Ismailia, Egypt

**Keywords:** Telescopic crown, Polyetheretherketone, Chromium alloys, Dental implants, Mandibular overdenture, Peri-implantitis

## Abstract

**Purpose:**

This pilot randomized clinical trial compared peri-implant tissue health around milled cobalt-chromium (CoCr) versus polyether ether ketone (PEEK) secondary telescopic crowns in implant-retained mandibular overdentures over 12 months.

**Methods:**

Twelve completely edentulous patients received two implants each. After randomization, secondary crowns were fabricated from milled CoCr (n = 6 patients) or PEEK (n = 6 patients). Assessor-blinded outcome assessments such as marginal bone loss (MBL), probing depth (PD), and plaque index (PI) were performed at baseline, 6, 9, and 12 months post-loading. Statistical analysis used the patient as the unit of analysis.

**Results:**

No statistically significant inter-group differences were found at any time point. At 12 months, mean MBL was 0.48 ± 0.15 mm for CoCr and 0.45 ± 0.16 mm for PEEK (*p* = 0.735). PD increased over time within groups but remained comparable between groups (CoCr: 2.58 ± 0.58 mm, PEEK: 2.33 ± 0.41 mm at 12 months; *p* = 0.411). PI scores also showed no significant inter-group differences (*p* > 0.05).

**Conclusions:**

Over 12 months, milled PEEK and CoCr secondary telescopic crowns demonstrated comparable peri-implant tissue health. PEEK presents a viable metal-free alternative for this application. These preliminary findings warrant confirmation in larger, long-term studies.

## Background

Implant-supported overdentures represent a well-established and reliable standard of care for the edentulous mandible [1–4]. The clinical success of these prostheses is profoundly influenced by the choice of attachment system. Telescopic crown attachments offer a compelling solution, providing predictable retention and stability while facilitating optimal peri-implant hygiene, a key factor in mitigating biological complications [5–8]. While numerous telescopic designs exist, a fundamental distinction lies in their biomechanical behavior. This stress-dissipating characteristic is of critical importance, as the long-term success of implant therapy is increasingly defined by the prevention of delayed biological complications, most notably peri-implantitis [8–12].

Beyond design, the material composition of the telescopic components is a paramount consideration with direct biological and mechanical implications. Cobalt-chromium (CoCr) alloys have been the benchmark for metal frameworks for decades, prized for their exceptional mechanical strength, precision of fit, and proven durability [11–15].

In contrast, polyetheretherketone (PEEK), a high-performance thermoplastic, has emerged as a viable alternative for prosthetic frameworks [13, 14, 16]. Its clinical appeal is partly rooted in its elastic modulus (~ 4 GPa), which is closer to that of cortical bone at the implant crest (approximately 15–25 GPa) than traditional rigid alloys or ceramics [14, 15]. This property may induce a beneficial cushioning effect, theoretically reducing stress concentration at the implant-abutment interface and the crestal bone region [16–22].

The elastic properties of restorative materials can influence how functional forces are transmitted to implants and surrounding bone [22–25]. Recent studies show that while all material pairings experience wear, the extent and pattern depend on the materials used [19]. Therefore, assessing PEEK-based attachments requires not only evaluating their short-term biological compatibility but also their capacity to sustain stable biomechanics and minimize retention loss over time. This pilot study was therefore designed to compare, over 12 months, the peri-implant tissue health, marginal bone loss, probing depth, and plaque accumulation, around CAD/CAM-milled PEEK versus cobalt-chromium secondary telescopic crowns in mandibular implant-retained overdentures.

Therefore, this clinical study aimed to compare the one-year peri-implant tissue response beneath mandibular implant-retained overdentures utilizing CAD/CAM-milled telescopic attachments. Specifically, we evaluated and compared marginal bone loss (MBL), probing depth (PD), and plaque index (PI) between secondary crowns fabricated from conventional CoCr and those fabricated from PEEK. The null hypothesis was that no significant differences would exist in MBL, PD, or PI between the two material groups.

## Methods

### Study design and registration

This study was a retrospective, assessor-blinded, parallel-group, pilot randomized controlled trial conducted at the Prosthodontics Department, Faculty of Dentistry, Minia University, Egypt. The protocol was approved by the Internal Review Board (IRB) “Institutional Ethical Committee” (Protocol registration number #528; at 01 November 2021) and registered retrospectively at [ClinicalTrials.gov] under identifier [NCT06637930] on 16 October 2024. Because registration occurred after recruitment began, the pre-specification of outcomes cannot be confirmed solely from the registry; however, all outcomes were defined in the protocol approved by the Institutional Ethical Committee (No. 528, 1 November 2021).The study was conducted in accordance with the Declaration of Helsinki and reported following the Consolidated Standards of Reporting Trials (CONSORT) guidelines (http://www.Consort-statement.org).

### Sample size and randomization

As a pilot RCT comparing novel (PEEK) and established (CoCr) materials, a formal power calculation was not performed. A sample size of six patients per group (12 implants per group) was chosen to provide preliminary data on feasibility, safety, and effect size for future definitive trials.

Randomization to the CoCr or PEEK group was performed in a 1:1 ratio using a computer-generated sequence (www.random.org). Allocation was concealed using sequentially numbered, opaque, sealed envelopes (SNOSE), opened by the laboratory technician only after the framework was scheduled for milling.

### Participants: eligibility, recruitment, and setting

**Fig. 1:**
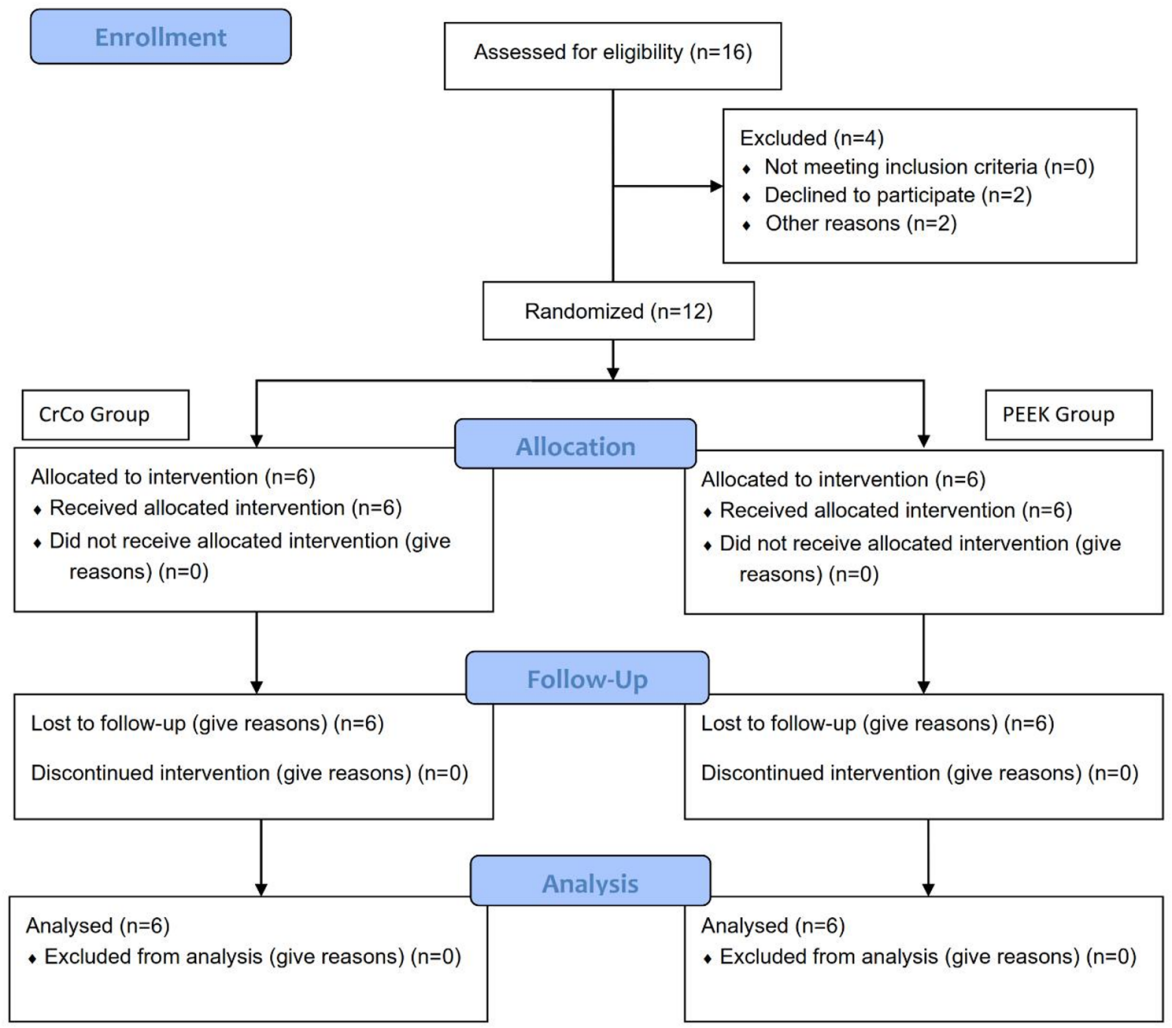
Flow diagram of the randomized clinical trial. Sixteen edentulous patients were assessed for eligibility; twelve were enrolled, received an implant, and were randomized to receive complete dentures with either cobalt-chromium (CoCr) or polyetheretherketone (PEEK) frameworks (n = 6 per group)

Eligible participants were systemically healthy, completely edentulous patients seeking implant-supported mandibular overdentures. Key inclusion criteria were: sufficient bone volume in the mandibular canine regions for 3.6 × 14 mm implants, a healed ridge (≥ 4 months post-extraction), and excluded contraindications for implant surgery (e.g., uncontrolled diabetes, bisphosphonate therapy, heavy smoking > 10 cigarettes/day). All patients provided an approved written informed consent. Recruitment occurred between 15 March 2022 and 11 September 2022 (Fig. 1).

### Interventions

**Fig. 2:**
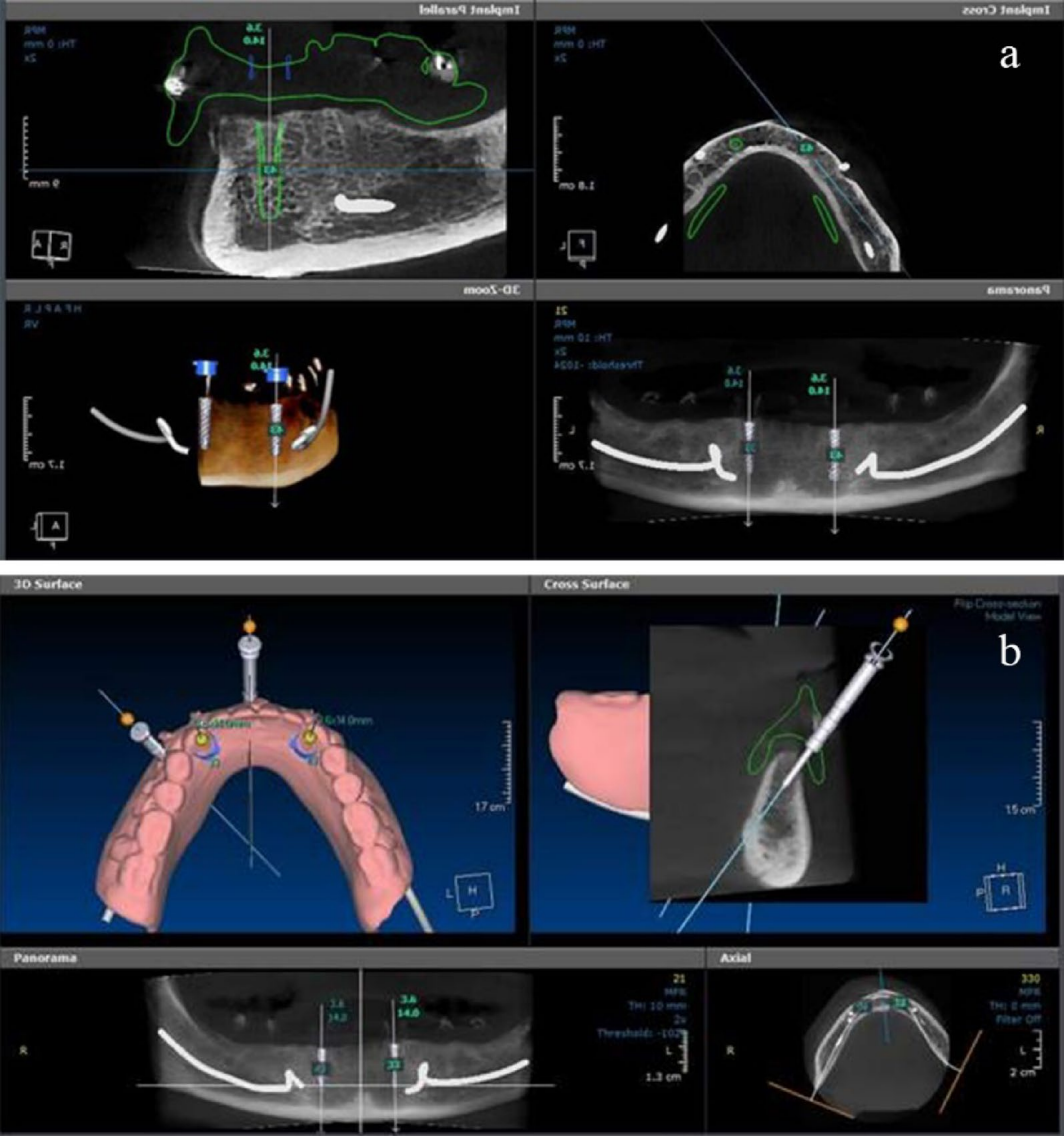
**a**, Preoperative CBCT-based digital planning of mandibular implant placement. Multiplanar views demonstrate implant angulation, depth, and relation to anatomical landmarks. **b**, Virtual guide design is shown to ensure prosthetically driven implant positioning.

Following a one-month adaptation period with new conventional complete dentures, tissue-supported surgical guides were designed via the computerized software (*In2Guide™, cybermed, Korea*) with 2 guiding holes (5 mm in diameter) for implant placement. They also had three anchor pin openings. The designed guides were fabricated from clear acrylic liquid resin (*E-Shell 450 Clear Resin liquid light polymerized, China)* and 3D printed via an in-office desktop 3D printer *(ETEC, Envisiontec GmbH, Gladbeck, Germany)*, Fig. 2.

All patients received two internal-connection implants (Superline II, Dentium, Korea; 3.6 × 14 mm) in the mandibular canine regions using a flapless, guided surgical protocol (Fig. 2). After a 3-month healing period, standard titanium conical primary crowns (abutments) with a 2° taper were CAD/CAM milled and torqued to 25 Ncm.

### Surgical procedures

**Fig. 3:**
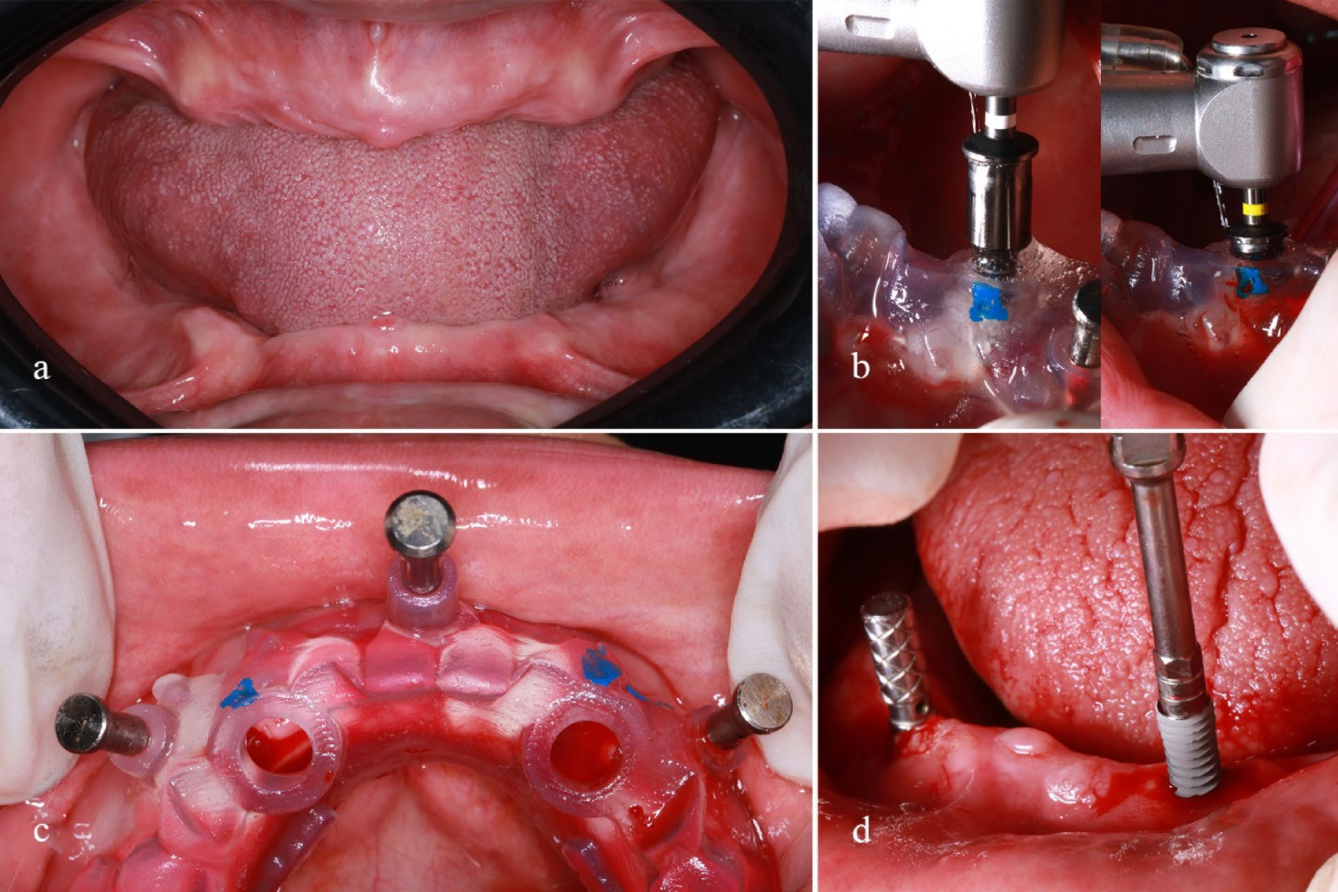
**a**, Intraoral view showing the edentulous mandibular arch before surgery. Soft-tissue condition and ridge morphology are demonstrated. **b**, Guided drilling protocol is initiated to the full length. **c**, Intraoperative placement of the surgical guide on the mandibular ridge before sleeve placement. Proper seating and stability of the guide are verified clinically after placing anchor pins. **d**, Clinical photograph showing free hand implant insertion in a previously prepared controlled osteotomy

The tissue-supported guide was then placed intraorally with anchor pins secured in the alveolar bone from the vestibular side. A tissue punch was used to remove soft tissue covering the osteotomy site. The osteotomy sites were then drilled sequentially via a standard bone drilling protocol (*Full Kit XGSFK, Dentium, Korea*) following the manufacturer’s guidelines. Finally, two root form implants were installed into the osteotomy sites and secured in place with a torque wrench (25 N) until the implants were flush with the bone; later, the cover screws were secured to the implant body, Fig. 3.

### Prosthetic procedures

**Fig. 4:**
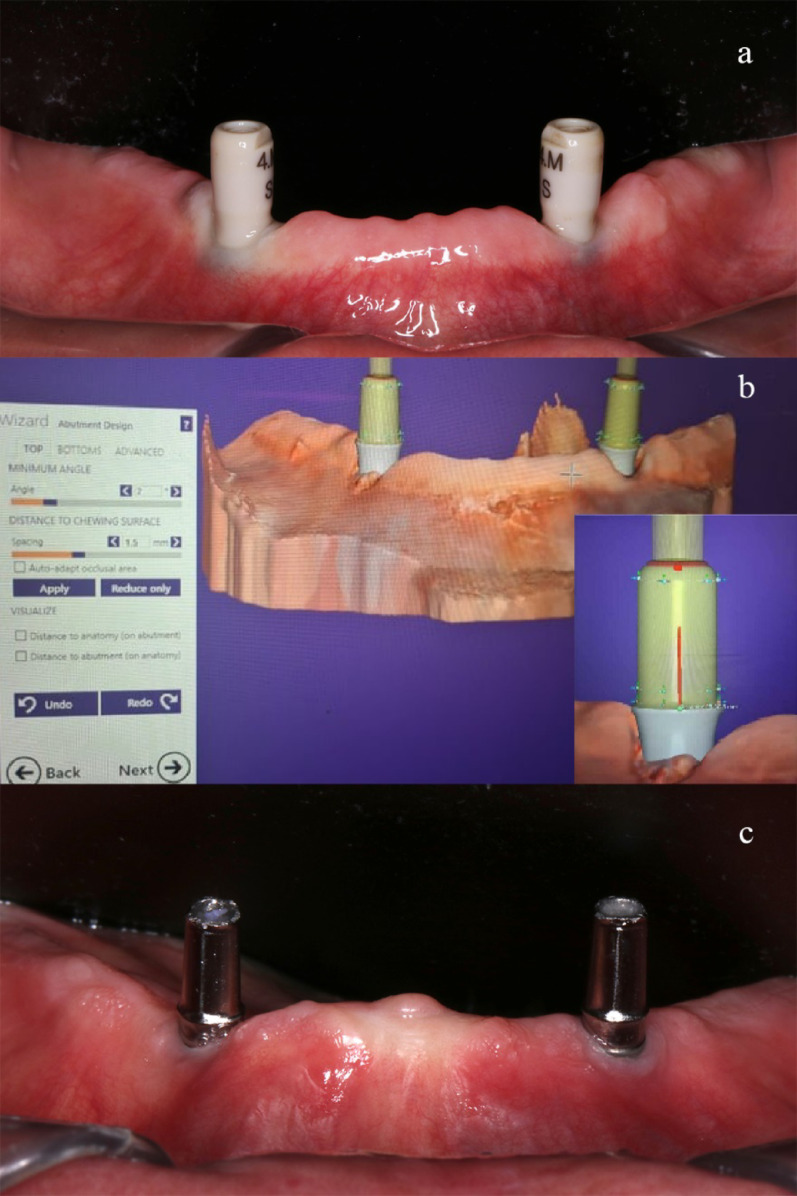
**a**, intraoral photograph showing Scan bodies before scanning to replicate implant positions. **b**, screenshot showing primary crowns design in Exocad keeping abutment parallelism. **c**, Customized primary crowns in place secured to the implant fixtures

After a 3-month healing period, digital intraoral implant scans were performed with 4 mm and 6 mm scan bodies (*HEX type Scan body, ART no. IHAB 40 06 H, Dentium, Korea*), Fig. 4a. The scanning was performed via an intraoral scanner (*Medit i500, MEDIT, Germany*), which created a 3D virtual model. Conical primary crowns (customized abutments) for telescopic crown attachment systems were designed via Exocad software (*DentalCAD Add-on Module, Germany).* The primary crowns were designed with a 2° taper and were 5–7 mm long. They also had a circular supragingival chamfer finish line with a width of 0.8–1 mm, Fig. 4b. The primary crowns were aligned and tightened to the implants at 25 Ncm torque and a periapical X-ray was performed to check the accurate fit, Fig. 4c.

Participants were then randomly allocated to one of two intervention groups:

**CoCr Group:** The overdenture framework with integrated secondary telescopic crowns was milled from a cobalt-chromium alloy block (Magnum H60, MESA ITALIA).

**PEEK Group:** The framework with integrated secondary telescopic crowns was milled from unfilled medical-grade PEEK (Glorious Dental Materials Co., Ltd., China; ISO 13485 certified; certified biocompatibility according to ISO 10993–5 and ISO 10993–10; elastic modulus ~ 4 GPa per manufacturer).

**Fig. 5:**
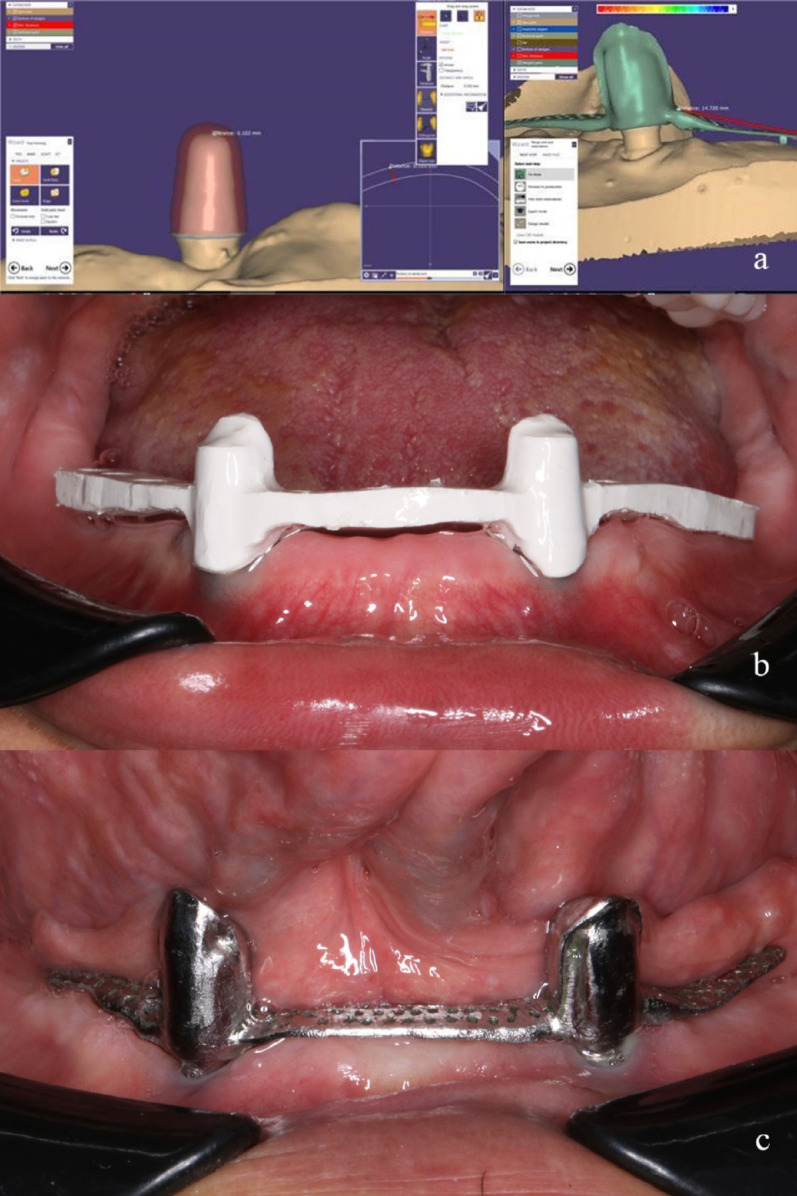
**a**, Screenshot from Exocad software showing designing and planning of the secondary crowns and their attached frameworks ready for milling. **b** and **c**, intraoral photographs representing PEEK and CoCr frameworks checked and verified

All secondary crowns were designed with a (100 µm) clearance fit (resilient design) and connected via a CAD/CAM-fabricated metal-reinforced framework to the denture base. The same experienced clinician delivered all prostheses, (Fig. 5).

The designed framework was sent to the milling center, where a 5-Axis dental milling machine (*ED5X V4.0, Emar, Egypt*) was used to mill the framework with integrated secondary crowns according to the grouping strategy in this study. Frameworks in the CoCr group were milled from CoCr alloy blocks, whereas those in the PEEK group were milled from PEEK blocks. The milled framework was checked intraorally for accuracy, fit, extension, and marginal fit of secondary crowns as well as physiologic relief. After verification of the framework, the intaglio surface of the existing conventional complete denture was relieved, and the framework was chairside picked up using autopolymerizing acrylic resin (Unifast Trad, GC, Japan), converting the denture into an implant-retained overdenture.

### Blinding (masking)

Due to the visual difference between materials, the treating prosthodontist and patient could not be blinded. However, critical blinding was maintained for outcome assessment. The radiologist analyzing MBL and the periodontal examiner assessing PD/PI were fully blinded to group allocation. Data was anonymized for statistical analysis.

### Outcomes (primary and secondary)

Primary Outcome: Mean marginal bone loss (MBL) per implant, measured radiographically at 12 months post-loading.

Secondary Outcomes: MBL at 6 and 9 months; peri-implant Probing Depth (PD) and modified Plaque Index (PI) at 0 (baseline), 6, 9, and 12 months.

### Clinical and radiographic assessment protocols

**Fig. 6:**
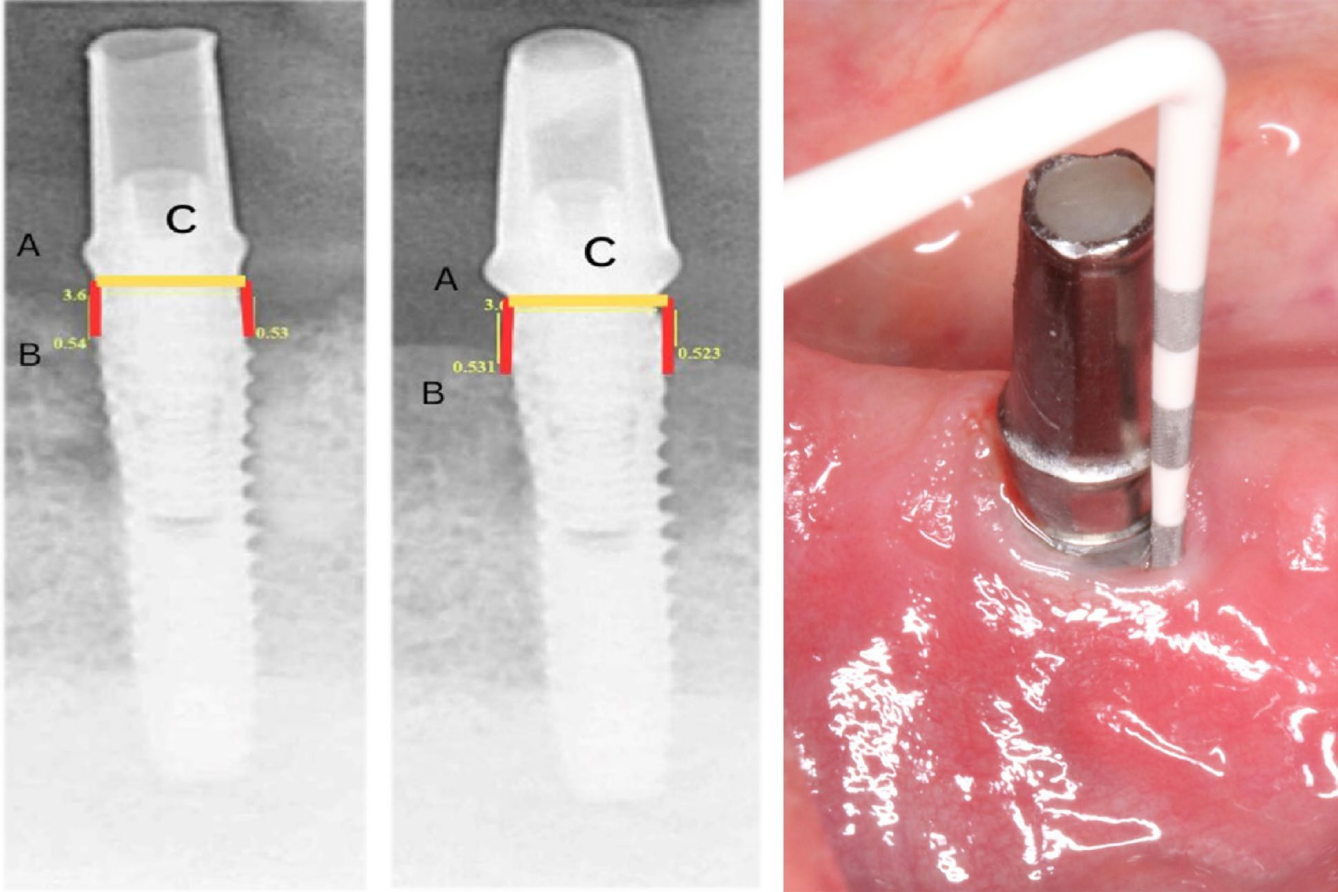
Post operative radiographic assessment of the MBL in both groups from the reference point to the bone level. **A**: represent the neck of the implant. **B**: represent first bone contact. **C**: represent calibration through implant width. Image on the right side for intraoral photograph showing pocket depth measuring using a single coded periodontal probe

*MBL*: Standardized, parallel-technique periapical radiographs were taken at loading and each follow-up using a customized index for reproducibility. Mesial and distal bone levels were measured from the implant neck to the first bone-to-implant contact using calibrated software (EZ Dent-i, VATECH). Measurements were performed in triplicate by a single calibrated examiner, with the mean used for analysis (Fig. 6).

*PD & PI*: PD was measured at four sites per implant using a pressure-controlled color-coded probe (*Williams Color-Coded Probe; Hu-Friedy, Chicago)*. PI was assessed using the modified Silness & Löe index (0–3). A single, calibrated periodontist performed all clinical exams (Fig. 6).

### Statistical analysis

Statistical analysis was performed using IBM SPSS v.23.0, with the patient as the statistical unit (implant-level data averaged per patient). Normality was assessed via Shapiro–Wilk tests. Parametric data (MBL, PD) were analyzed using repeated-measures ANOVA (within-subjects: time; between-subjects: group) with Bonferroni post-hoc tests. Non-parametric PI scores were analyzed using the Friedman and Mann–Whitney U tests. The significance level was set at *p* ≤ 0.05.

## Results

This study included twelve participants, consisting of nine males (75%) and 3 females (25%). The median age of the patients was 58 years, with an age range of 52–64 y. All participants underwent identical surgical, prosthetic, and assessment procedures. Throughout the study, none of the twenty-four implants showed any signs or symptoms of failure throughout the evaluation period (100% implant success rate).

### Marginal bone loss (mm)

#### Mesial side

After six, nine and 12 months, there was no statistically significant difference between the two groups (*P*-value = 0.328, Effect size = 0.096), (*P*-value = 0.747, Effect size = 0.011) and (*P*-value = 0.831, Effect size = 0.005), respectively.

In both groups, there was a statistically significant change in mesial MBL by time (*P*-value = 0.007, Effect size = 0.664) and (*P*-value = 0.001, Effect size = 0.807), respectively. Pair-wise comparisons between time points revealed that there was no statistically significant change in MBL from six to nine months, followed by a statistically significant increase in mesial MBL from nine to 12 months.

#### Distal side

After six, nine and 12 months, there was no statistically significant difference between the two groups (*P*-value = 0.654, Effect size = 0.021), (*P*-value = 0.789, Effect size = 0.005) and (*P*-value = 0.735, Effect size = 0.012), respectively.

In the CoCr group, there was a statistically significant change in distal MBL by time (*P*-value = 0.001, Effect size = 0.815). Pair-wise comparisons between time points revealed that there was no statistically significant change in distal MBL from six to nine months, followed by a statistically significant increase in distal MBL from nine to 12 months. In the PEEK group, there was a statistically significant change in distal MBL by time (*P*-value = 0.003, Effect size = 0.727). Pair-wise comparisons between time points revealed that there was a statistically significant increase in distal MBL from six to nine months, as well as from nine to 12 months.

#### Mean of the two sides (overall marginal bone loss)

The overall mean MBL showed no statistically significant difference between groups at 6 months (CoCr: 0.42 ± 0.15 mm, PEEK: 0.35 ± 0.15 mm; *p* = 0.478), 9 months (CoCr: 0.44 ± 0.15 mm, PEEK: 0.41 ± 0.18 mm; *p* = 0.767), or 12 months (CoCr: 0.48 ± 0.15 mm, PEEK: 0.45 ± 0.16 mm; *p* = 0.735).

In the CoCr group, there was a statistically significant change in MBL by time (*P*-value < 0.001, Effect size = 0.828). Pair-wise comparisons between time points revealed that there was no statistically significant change in MBL from six to nine months, followed by a statistically significant increase in MBL from nine to 12 months.

**Fig. 7:**
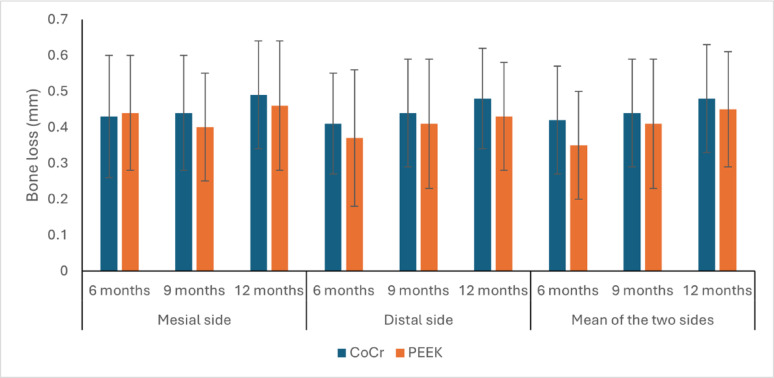
Bar graph comparing marginal bone loss (in mm) over a 12-month period between implant-supported complete dentures with cobalt-chromium (CoCr) and polyetheretherketone (PEEK) frameworks

In the PEEK group, there was a statistically significant change in MBL by time (*P*-value < 0.001, Effect size = 0.87). Pair-wise comparisons between time points revealed that there was a statistically significant increase in MBL from six to nine months, as well as from 9 to 12 months (Table 1, Fig. 7).

**Table 1:** Descriptive statistics, results of repeated measures ANOVA test for comparison between marginal bone loss (mm) in the two groups and the changes within each group

Site	Time	CoCr (n = 6)		PEEK (n = 6)		*P*-value	Effect size (Partial Eta Squared)
		Mean	SD	Mean	SD		
Mesial	6 months	0.43 ^b^	0.17	0.44 ^b^	0.16	0.328	0.096
	9 months	0.44 ^b^	0.16	0.4 ^b^	0.15	0.747	0.011
	12 months	0.49 ^a^	0.15	0.46 ^a^	0.18	0.831	0.005
	*P*-value	0.007		0.001*			
	*Effect size (Partial Eta Squared*	0.664		0.807			
Distal	6 months	0.41 ^b^	0.14	0.37 ^c^	0.19	0.654	0.021
	9 months	0.44 ^b^	0.15	0.41 ^b^	0.18	0.789	0.008
	12 months	0.48 ^a^	0.14	0.43 ^a^	0.15	0.637	0.023
	*P*-value	0.001		0.003*			
	*Effect size (Partial Eta Squared*	0.815		0.727			
Mean of the two sides	6 months	0.42 ^b^	0.15	0.35 ^c^	0.15	0.478	0.052
	9 months	0.44 ^b^	0.15	0.41 ^b^	0.18	0.767	0.009
	12 months	0.48 ^a^	0.15	0.45 ^a^	0.16	0.735	0.012
	*P*-value	< 0.001		< 0.001*			
	*Effect size (Partial Eta Squared*	0.828		0.87			

* Significant at P ≤ 0.05, Different superscripts in the same column indicate statistically significant change by time

### Pocket depth (PD) in mm

At baseline, the mean PD was 1.50 ± 0.45 mm in the CoCr group and 1.58 ± 0.38 mm in the PEEK group, with no statistically significant inter-group difference (*p* = 0.734). This non-significant difference persisted at 6 months (CoCr: 1.83 ± 0.52 mm, PEEK: 1.83 ± 0.41 mm; *p* = 1.000), 9 months (CoCr: 2.25 ± 0.52 mm, PEEK: 1.92 ± 0.38 mm; *p* = 0.235), and 12 months (CoCr: 2.58 ± 0.58 mm, PEEK: 2.33 ± 0.41 mm; *p* = 0.411).

Regarding changes over time in the CoCr group, there was a statistically significant change in PD by time (*P*-value < 0.001, Effect size = 0.944). Pair-wise comparisons between time points revealed that there was a statistically significant increase in PD after six months, from six to nine, as well as from 9 to 12 months.

**Fig. 8:**
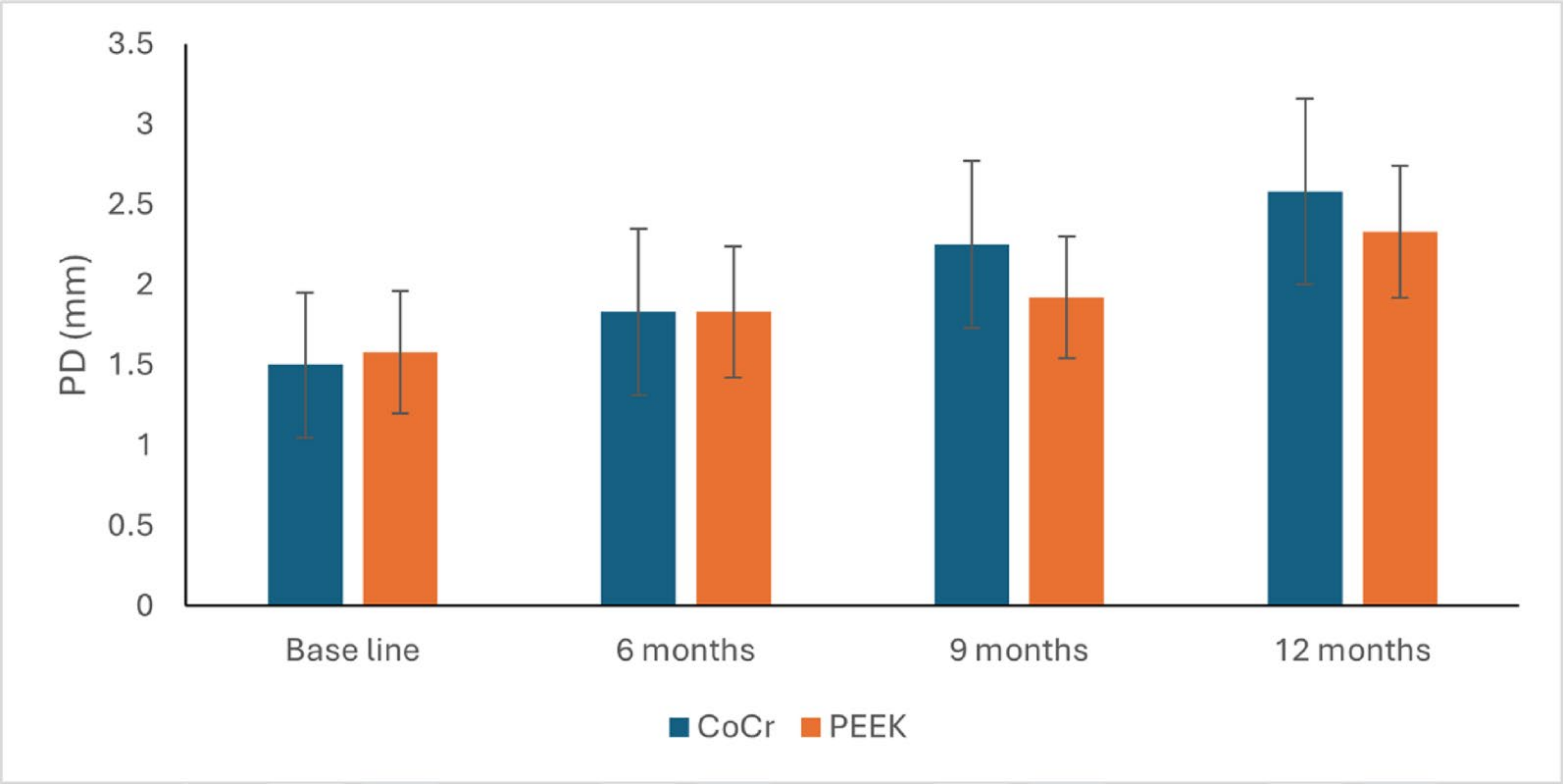
Line graph depicting probing depth (PD in mm) changes from baseline through 12 months of follow-up for the CoCr and PEEK framework groups. The plot illustrates the periodontal health parameter trajectory around the supporting implant for each material cohort at 6-, 9-, and 12-month time points

While for the changes over time in the PEEK group, there was a statistically significant change in PD by time (*P*-value = 0.001, Effect size = 0.869). Pair-wise comparisons between time points revealed that there was a statistically significant increase in PD after six months, which was followed by a non-significant statistical change in PD from six to nine months. From nine to 12 months, there was a statistically significant increase in PD, Table 2, Fig. 8.

**Table 2:** Descriptive statistics, results of repeated measures ANOVA test for comparison between PD (mm) in the two groups and the changes within each group

Time	CoCr (n = 6)		PEEK (n = 6)		*P*-value	Effect size (Partial Eta Squared)
	Mean	SD	Mean	SD		
Base line	1.5 ^d^	0.45	1.58 ^c^	0.38	0.734	0.012
6 months	1.83 ^c^	0.52	1.83 ^b^	0.41	1	0
9 months	2.25 ^b^	0.52	1.92 ^b^	0.38	0.235	0.138
12 months	2.58 ^a^	0.58	2.33 ^a^	0.41	0.411	0.069
*P*-value	< 0.001*		0.001*			
Effect size (Partial Eta Squared)	0.944		0.869			

* Significant at P ≤ 0.05, Different superscripts in the same column indicate statistically significant change by time

### Plaque index (PI)

At baseline, the median PI score was 0 (range 0–0) in both groups (*p* = 1.000). At 6 months, the median score was 0.5 (range 0–1; mean 0.50 ± 0.55) in the CoCr group and 0 (range 0–1; mean 0.17 ± 0.41) in the PEEK group, with no significant inter-group difference (*p* = 0.241). This pattern continued at 9 months (CoCr median: 1, range 0–1, mean 0.83 ± 0.41; PEEK median: 1, range 0–1, mean 0.67 ± 0.52; *p* = 0.523) and at 12 months (CoCr median: 1, range 0–2, mean 1.00 ± 0.63; PEEK median: 1, range 0–1, mean 0.83 ± 0.41; *p* = 0.598).

Both groups showed a statistically significant change in PI scores over time (CoCr: *p* = 0.008, Effect size = 0.653; PEEK: *p* = 0.010, Effect size = 0.63). Pair-wise comparisons within each group revealed no statistically significant change from baseline to 6 months, followed by a statistically significant increase from 6 to 9 months. No further statistically significant change was observed from 9 to 12 months (Table 3).

**Table 3:** Descriptive statistics, results of Mann–Whitney U test for comparison between PI scores in the two groups and Friedman’s test for the changes within each group

Time	CoCr (n = 6)		PEEK (n = 6)		*P*-value	Effect size (d)
	Median (Range)	Mean (SD)	Median (Range)	Mean (SD)		
Base line	0 (0, 0) ^b^	0 (0)	0 (0, 0) ^b^	0 (0)	1	0
6 months	0.5 (0, 1) ^b^	0.5 (0.55)	0 (0, 1) ^b^	0.17 (0.41)	0.241	0.577
9 months	1 (0, 1) ^a^	0.83 (0.41)	1 (0, 1) ^a^	0.67 (0.52)	0.523	0.28
12 months	1 (0, 2) ^a^	1 (0.63)	1 (0, 1) ^a^	0.83 (0.41)	0.598	0.233
*P*-value	0.008		0.010			
Effect size (w)	0.653		0.63			

*: Significant at P ≤ 0.05, Different superscripts in the same column indicate statistically significant change by time

## Discussion

This retrospective pilot randomized controlled trial provides the first comparative clinical data on peri-implant tissue health around CAD/CAM-milled PEEK versus conventional CoCr secondary telescopic crowns for implant-retained overdentures. The primary finding, the absence of statistically significant differences in marginal bone loss (MBL), probing depth (PD), or plaque index (PI) over 12 months, supports the initial null hypothesis. Critically, all parameters remained within ranges indicative of peri-implant health, suggesting that PEEK is a biologically viable alternative to CoCr for this specific application within the studied timeframe.

The comparable MBL between groups (CoCr: 0.48 ± 0.15 mm; PEEK: 0.45 ± 0.16 mm) is clinically reassuring. These values align with the established success criterion of < 2.0 mm bone loss in the first year of loading and are consistent with outcomes reported for other well-designed telescopic attachment systems [26]. This bone preservation is likely multifactorial, attributable to the study’s standardized protective protocol: flapless guided surgery minimized trauma, delayed loading ensured osseointegration, and supragingival abutment finish lines facilitated hygiene [27, 28]. Although overall MBL did not differ between groups, the time-course analysis revealed a slightly different pattern: the PEEK group exhibited significant bone loss during both the 6–9 and 9–12 month intervals, while the CoCr group reached significance only between 9–12 months. The clinical implications of this temporal divergence are unclear given the small sample size and the absence of a final inter-group difference, but it may reflect subtle differences in early bone remodeling and warrants monitoring in future studies.

It has been hypothesized that PEEK’s lower elastic modulus (~ 4 GPa), closer to that of cortical bone, might confer a biomechanical advantage by damping functional forces. However, the present study was not designed to isolate the effect of elastic modulus, and the absence of significant inter-group differences cannot be interpreted as evidence for or against this hypothesis. One possible interpretation, requiring dedicated investigation, is that the 100 µm resilient clearance already provided a level of shock absorption that masked any additional effect of the secondary crown material. Regardless, the comparable clinical outcomes suggest that factors such as prosthetic design, occlusal scheme, and plaque control may be more immediate determinants of early peri-implant health than the material of the secondary crown.

The parallel trajectories of PD and PI in both groups reflect a typical early adaptive and maintenance phase. The stable, shallow PDs (< 3 mm) and absence of suppuration are strong indicators of peri-implant mucosal health, as defined by current classification systems [23]. The significant rise in PI from baseline in both groups underscores a universal clinical challenge: maintaining favorable plaque control around any supra-implant prosthesis. These finding redirects focus from material type to prosthesis design and patient-specific hygiene protocols as key element for long-term health.

Our data contribute to the unresolved debate on material-specific biofilm affinity. While in vitro studies, such as that by Abd El Azeem et al. [29], report higher bacterial adhesion on PEEK, our clinical findings over 12 months did not show a corresponding negative clinical impact (e.g., increased inflammation or PD). This discrepancy between in vitro adhesion studies and in vivo clinical outcomes is well-documented and may be attributed to the profound modifying influence of the salivary pellicle, host immune factors, and oral hygiene practices in the clinical environment. It suggests that surface free energy or roughness in vitro may not be reliable standalone predictors of long-term clinical peri-implant health.

Biological equivalence permits evidence‑based material selection guided by patient‑specific and technical factors. PEEK is indicated for metal‑free requirements (allergy, esthetics, thin biotypes) and may reduce abrasion of titanium abutments due to its lower wear hardness. CoCr remains preferred when long‑term proven durability and cost‑effectiveness are priorities, supported by decades of validation. This shifts the paradigm from seeking a biologically “superior” material to selecting the “most appropriate” one based on comprehensive assessment.

A key strength of this study is its RCT design, incorporating blinded outcome assessment, which minimizes bias in parameter measurement. The use of CAD/CAM for both materials ensures a high and comparable standard of fit, isolating “material” as the primary variable.

The limitations must be interpreted through the explicit lens of a pilot feasibility study [CONSORT Extension for Pilot Trials]. The small sample size (n = 6 per group) is appropriate for generating preliminary safety and effect size data but is underpowered to detect small, potentially clinically relevant differences. Therefore, our results indicate “no evidence of a difference” rather than definitive “evidence of no difference”. The twelve-month period is adequate to assess early biological response but is insufficient to evaluate long-term bone remodeling, material degradation, or late-onset peri-implantitis. Retrospective trial registration, while suboptimal, does not invalidate the internally collected data but underscores the importance of prospective registration for future definitive work. A further limitation is the absence of mechanical outcome data, including retention force, wear of the secondary crown intaglio, and component fractures. PEEK’s viscoelastic behavior may influence long-term retention and wear patterns differently than CoCr. Therefore, the clinical stability of the attachment cannot be fully evaluated from this study. This gap should be addressed in future trials with longer follow-up.

Within pilot study constraints, CAD/CAM‑milled PEEK and CoCr secondary crowns demonstrated comparable 12‑month peri‑implant health. PEEK is a clinically acceptable metal‑free alternative. These findings support feasibility and rationale for a definitive, adequately powered non‑inferiority RCT incorporating patient‑reported outcomes and technical complications (retention loss, wear, fracture) over ≥ 3 years to establish material selection guidelines. Future studies should include larger samples, mechanical wear evaluation, and extended follow‑up.

## Conclusion

This pilot randomized controlled trial provides the first clinical evidence that CAD/CAM-milled PEEK secondary telescopic crowns represent a biologically viable alternative to the conventional CoCr standard for two-implant-retained mandibular overdentures. Over 12 months, no statistically significant differences in marginal bone loss, probing depth, or plaque accumulation were observed between CAD/CAM-milled PEEK and CoCr secondary telescopic crowns; all parameters remained within clinically acceptable ranges.

## Data Availability

No datasets were generated or analysed during the current study.
